# Portable vs. Benchtop NIR-Sensor Technology for Classification and Quality Evaluation of Black Truffle

**DOI:** 10.3390/molecules27030589

**Published:** 2022-01-18

**Authors:** Christoph Kappacher, Benedikt Trübenbacher, Klemens Losso, Matthias Rainer, Günther K. Bonn, Christian W. Huck

**Affiliations:** 1 Institute of Analytical Chemistry and Radiochemistry, Leopold-Franzens University Innsbruck, 6020 Innsbruck, Austria; christoph.kappacher@uibk.ac.at (C.K.); Benedikt.Truebenbacher@student.uibk.ac.at (B.T.); Klemens.Losso@uibk.ac.at (K.L.); M.Rainer@uibk.ac.at (M.R.); Günther.Bonn@uibk.ac.at (G.K.B.); 2 ADSI—Austrian Drug Screening Institute GmbH, 6020 Innsbruck, Austria

**Keywords:** black truffle, near-infrared spectroscopy, chemometrics, food quality, food adulteration

## Abstract

Truffles represent the best known and most expensive edible mushroom. Known as *Ascomycetes*, they belong to the genus *Tuber* and live in symbiosis with plant host roots. Due to their extraordinary taste and smell, truffles are sold worldwide for high prices of up to 3000–5000 euros per kilogram (*Tuber magnatum* PICO). Amongst black truffles, the species *Tuber melanosporum* VITTAD. is highly regarded for its organoleptic properties. Nonetheless, numerous different sorts of black truffle are offered at lower prices, including *Tuber aestivum* VITTAD., *Tuber indicum* and *Tuber uncinatum*, which represent the most frequently consumed types. Because truffles do not differ visually for inexperienced consumers, food fraud is likely to occur. In particular, for the highly prized *Tuber melanosporum*, which morphologically forms very similar fruiting bodies to those of *Tuber indicum*, there is a risk of fraud via imported truffles from Asia. In this study, 126 truffle samples belonging to the four mentioned species were investigated by four different NIR instruments, including three miniaturized devices—the Tellspec Enterprise Sensor, the VIAVI solutions MicroNIR 1700 and the Consumer Physics SCiO—working on different technical principles. Three different types of measurement techniques were applied for all instruments (outer shell, rotational device and fruiting body) in order to identify the best results for classification and quality assurance in a non-destructive manner. Results provided differentiation with an accuracy up to 100% for the expensive *Tuber melanosporum* from *Tuber indicum*. Classification between *Tuber melanosporum*, *Tuber indicum*, *Tuber aestivum* and *Tuber uncinatum* could also be achieved with success of 100%. In addition, quality monitoring including discrimination between fresh and frozen/thawed, and prediction of the approximate date of harvesting, was performed. Furthermore, feasibility studies according to the geographical origin of the truffle were attempted. The presented work compares the performance for prediction and quality monitoring of portable vs. benchtop NIR devices and applied measurement techniques in order to be able to present a suitable, accurate, fast, non-destructive and reliable method for consumers.

## 1. Introduction

Food fraud occurs globally in many kinds of food products, including most common food categories such as olive oil [1], fish [2], milk [3] and coffee [4]. Expensive and high-quality premium products, such as meat [5], spices [6], honey [7,8] and truffles [9], are particularly at risk due to the large profit margin. In addition to minimizing the risk to health if toxic substances are applied for adulteration, a means of developing high-quality products with verifiable origin and quality is desired. Food fraud scandals, such as the addition of melamine in baby formula in China [10] or the European horse-meat scandal [11], have increased the pressure on food laboratories to investigate and explore fast screening methods for the detection of food adulteration. Spectroscopic methods are ideally suited for this purpose as they enable non-destructive, fast, green and reliable measurements [12,13,14,15,16,17,18]. These techniques are already applied in a wide variety of industry related areas including agriculture, cosmetics, pharmaceutical production, the textile industry and petroleum processing [19]. Recent developments in the direction of portable and miniaturized devices, in combination with appropriate software algorithms provided by manufacturers, have enabled the possibility for quality assessment by consumers to meet the demand for self-verifiable quality [14,19]. For this purpose, manufacturers provide a large number of miniaturized portable devices including: Thermo Fischer Scientific’s microPHAZIR; Texas Instruments’ NIRscan; Spectral Engines’ NIRONE sensors; Si-Ware Systems’ NeoSpectra scanner; SouthNest Technology’s nanoFTIR NIR; and the devices used in this study namely: the VIAVI solutions MicroNIR, the Consumer Physics SCiO and the Tellspec Enterprise Sensor [15,16,20].

Due to the strong organoleptic similarity of highly prized *T. melanosporum* and much cheaper *T. indicum*, food adulteration is difficult to observe by the naked eye for unexperienced consumers. Due to the above-mentioned cases of food adulteration, many analytical methods for identification and confirmation of black truffle have been applied, including matrix assisted time of flight mass spectrometry (MALDI-TOF-MS) [21], stable isotope ratio analysis [22], headspace solid-phase microextraction followed by gas chromatography-mass spectrometry (HS-SPME/GC-MS) [23], capillary gel electrophoresis and real-time PCR [24], inductively coupled plasma mass spectrometry [25], and FT-NIR spectroscopy and chemometrics with freeze-dried samples [26]. Destructive and cost-intensive sample preparation, expensive equipment and highly trained personnel, and a huge time effort are required for the presented methods. In 2020, Segelke et al. reported on species classification by FT-NIR and chemometrics for differentiation within white and black truffle species [26]. Although they reported successful differentiation between black truffle species, the presented method is not suitable for consumers due to the need to lyophilize and grind truffles before measurement. The obtained results presented in the current study can be applied by anybody without requiring special training, and offers rapid and nondestructive discrimination between truffle species, in addition to quality assurance.

Freezing is frequently used for long-term storage of *Tuber* species [27]. However, Culleré et al. revealed a significant drop in quality for *T. melanosporum* as a consequence of the freezing process [28]. Changes in 15 aromatic compounds were detected over periods of 1, 20, 40 and 60 days using headspace solid phase microextraction followed by gas chromatography and mass spectrometry analysis (HS-SPME-GC-MS). Results revealed a significant change in the volatile composition of truffles, explaining the loss of freshness within 24 h after freezing [28]. In 2018, Marco et al. compared different long-term preservation treatments of black truffle including freezing and lyophilization. By the use of solid phase micro extraction (SPME) prior to gas chromatography-olfactometry and sensory descriptive aroma analysis they presented similar results regarding loss of quality [27]. As a consequence, discrimination between fresh and frozen/thawed samples was investigated in order to ensure verifiable quality for this highly prized product.

Although truffles are sold globally, they are harvested within a small geographical space. Transport and distribution through traders are related to a certain amount of time and, thus, a loss of quality. An attempt was made to identify a connection between near infrared spectral information and the date of harvest.

In this study, we investigated 126 samples of four truffle species, namely, *Tuber aestivum*, *Tuber indicum*, *Tuber melanosporum* and *Tuber uncinatum*. All samples were investigated using four NIR devices, including one benchtop and three portable devices. Three different measurement techniques were applied to all spectrometers. Spectral information was processed using chemometrics to create reliable models for correct identification of species and, furthermore, to make a statement about the quality of the truffle. Results were evaluated and compared in order to determine the differences in portable vs. benchtop NIR devices.

## 2. Results

### 2.1. Visual Spectral Exploration and Investigation

**Figure 1:**
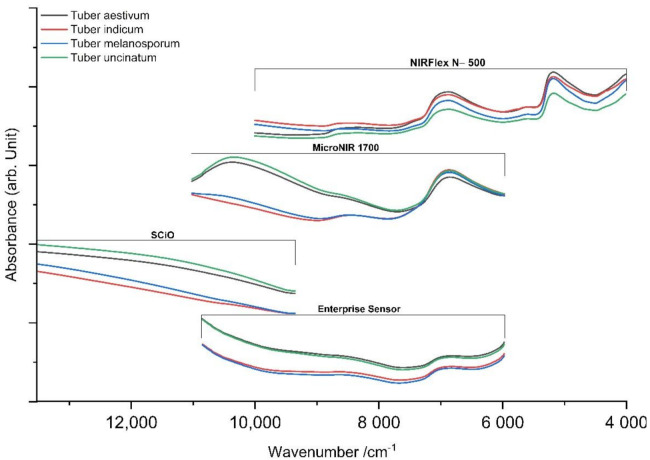
Mean value for recorded spectra (log1/R for NIRFlex N-500, SCiO, Enterprise Sensor) of the outer skin (method 1) compared for all four devices.

Figure 1 displays the mean of all untreated absorbance spectra of method 1 (log1/R for NIRFlex N-500, SCiO and Enterprise Sensor), namely, *T. aestivum*, *T. indicum*, *T. melanosporum* and *T. uncinatum*. It can be observed that *T. indicum* and *T. melanosporum* can be separated from *T. aestivum* and *T. uncinatum* by the naked eye, especially in the range of higher wavenumbers. Nonetheless, classification between these two groups is hardly feasible without spectroscopic pretreatment and chemometric analysis.

The obtained spectra showed high intensity deviations covering the entire spectrum due to scattering effects caused by the different textures of the samples. Additionally, the rounded geometry of truffles led to further scattering, although we attempted to record planes that were as flat as possible for measurements of theperidium. Analysis of scattering effects through descriptive statistics led to the conclusion that they mostly consist of wavelength-independent effects induced by the sample texture considered to be additive scattering effects.

The resulting spectra of all samples mostly contained broad and overlapping bands caused by the main constituents of the truffle, including proteins (7.4–12.9 g/100 g), carbohydrates (0.15–7.13 g/100 g) and lipids and water (73.8–76.4%) [29,30]. High absorption regions were found in wavelength regions of 6897 cm^−1^ (O-H stretching, first overtone) and 5155 cm^−1^ (O-H stretching and deformation) caused by water, overlapping signals of carbohydrates in fresh truffles [26]. Between the mentioned regions, at around 6670 cm^−1^ a broad band induced by N-H stretching (first overtone) of proteins occurs, although this declines through nearby water bands. Double peaks at 4340 and 4260 cm^−1^ of carbohydrates (CH_2_− symmetric and asymmetric stretching) are hard to observe for spectra of the peridium, but could be identified for measurements of the gleba [31]. This also applies to further CH-stretching (first overtone) and CH_2_− vibrations (around 5760 and 5740 cm^−1^) assigned to carbohydrates which could be identified [31]. Spectral regions between 4400 and 4200 cm^−1^, representing the lipid content of truffles, were only observed for NIRFlex N-500. Absorbance peaks with lower intensities observed around 8150 to 8750 cm^−1^ relies on the second overtone of the C-H stretching and can be attributed to fatty acids and lipids. Wavelengths above approximately 9000 cm^−1^, which is in close proximity to the visual region, showed a high diversity between truffle sorts caused by the different color of the truffle flesh (gleba), although the peridiumcan be hardly discriminated by the naked eye.

**Figure 2:**
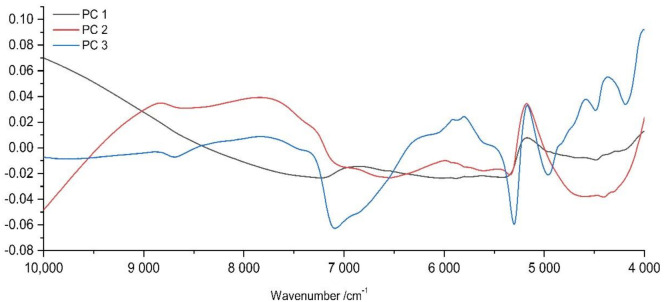
Loading plots of Büchi NIRFlex N-500 spectra after log1/R, SNV, Savitzky–Golay derivative and reduction showing the first three principal components for principal component analysis of all truffle samples using method 1.

In the recent work by Segelke and Schelm et al., water bands were excluded for analysis of lyophilized truffles as they overlap bands of proteins and carbohydrates and, therefore, superimpose the information beneath [26]. For this study, full spectral information, including water bands, was utilized. This is caused by the content of water, which differs within *Tuber* species, having values of 74.8%, 76.4% and 73.8% for *T. melanosporum*, *T. aestivum* and *T. indicum*, respectively [30].Therefore, it can be concluded that water bands in the spectrum should not be excluded as they might provide useful information for subsequent classification. This fact is supported by the loading plots of PCA (Figure 2). This plot for Büchi NIRFlex N-500 visualizes a major contribution to the prediction model by water bands (spectral pretreatment log1/R, SNV, Savitzky–Golay derivative and reduction) especially for principal components 2 and 3 at about 5155 and 6897 cm^−1^, corresponding to OH-stretching vibrations.

### 2.2. Classification and Quality Assurance of Black Truffle

From the first step, we now report the results for classification of the fresh truffles *T. melanosporum*, *T. indicum*, *T. aestivum* and *T. uncinatum*; differentiation between *T. melanosporum* and *T. indicum*; and, furthermore, evaluate the quality status of truffles, including discrimination between fresh and frozen-thawed truffles and the geographical origin.

#### 2.2.1. Classification of Fresh Truffle

**Figure 3:**
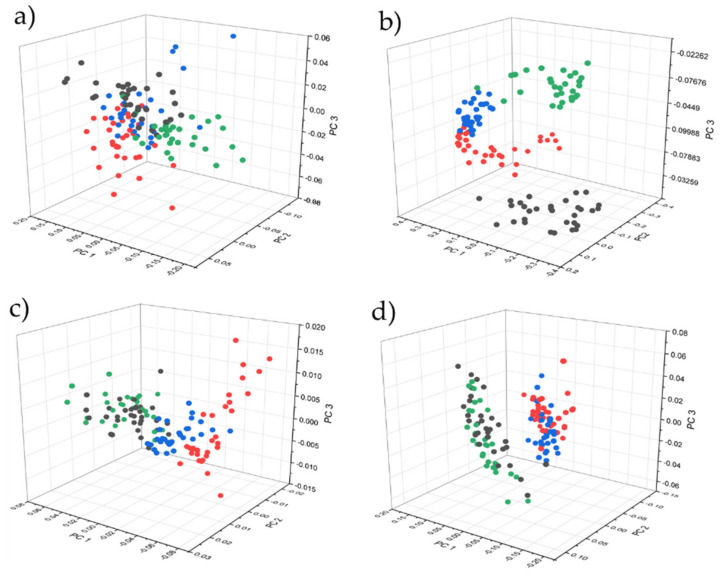
PCA scores 3D-plot of pretreated spectra (according to Table 5) of *T. aestivum* (black), *T. indicum* (red), *T. melanosporum* (blue) and *T. uncinatum* (green) for (**a**) Büchi NIRFlex N-500, (**b**) VIAVI solutions MicroNIR 1700, (**c**) Consumer Physics SCiO and (**d**) Tellspec Enterprise Sensor.

Unsupervised classification was performed for the complete pretreated dataset by applying the PCA algorithm to visually evaluate spectral differences of the sample set (Figure 3). Samples of the entire dataset were subsequent divided into calibration (84 samples) and validation (42 samples) sets by applying Kennard–Stone sample selection. A calibration set was used for LDA model construction by applying 10 principal components and the Mahalanobis method, and further used for predicting the validation set.

Figure 3 illustrates the score plots for the acquired spectra of method 1 for all applied devices after spectral pretreatment. Hereby, only results for method 1 are visually represented and discussed because this method represents a suitable method, applicable for customers without the use of additional equipment (rotational device) and the possibility of non-invasive measurements.

The plot for NIRFlex N-500 (Figure 3a shows no clear separation of sample classes, although the best results were achieved with 100% accuracy for the validation set as presented in Table 1. Furthermore, MicroNIR 1700 visually reveals a highly separated cluster of points with slightly overlapping samples, but nonetheless is not able to predict with high accuracy for supervised classification (LDA), correctly predicting 38 of 42 samples. The prediction performance of SCiO, which correctly predicted 38 of 42 samples, was surprising, although only 12 resolution elements were used for spectral recording. The worst results were achieved by the Enterprise Sensor, which correctly predicted only 33 of 42 samples. *T. aestivum* and *T. uncinatum* were clearly separated from *T. indicum* and *T. melanosporum*, although discrimination within these two groups was achieved with little success.

In the next section, a detailed discrimination between *T. melanosporum* and *T. indicum* is presented.

#### 2.2.2. Discrimination between *T. melanosporum* and *T. indicum*

As a result of the food fraud in which cheap *T. indicum* was sold as expensive *T. melanosporum*, a supervised classification model (LDA) was built using a dataset comprising these two classes. Similarly, samples were split into calibration (44 samples) and validation (21 samples) sets using Kennard–Stone sample selection by applying the same ratio.

As can be observed in Table 2, classification was achieved with highly accurate results throughout all devices and methods. For measurements of the outer skin (method 1), three of the four devices were able to predict the species of truffle with an accuracy of 100% in relation to the independent sample set. Measurements on the rotational plate were unexpectedly worse, achieving 100% for NIRFlex N-500, 100% for MicroNIR 1700 and 95.2% for SCiO. The Enterprise Sensor was able to correctly identify 90.5% for method 1, 85.7% for method 2 and 100% for the destructive method 3.

#### 2.2.3. Quality Evaluation—Discrimination between Fresh and Frozen/Thawed Truffle

The performance of NIR devices to discriminate between fresh and frozen/defrosted truffle samples was tested by applying measurements of the outer skin (method 1) using all fresh and 15 frozen/thawed samples of each *Tuber* species (distribution selected by Kennard–Stone sample selection). Spectral pretreatment was performed according to Table 5. LDA was executed on the calibration set using the Mahalanobis method and by applying five components. Table 3 represents the results obtained for the corresponding NIR devices.

#### 2.2.4. Quality Evaluation—Aging Studies of *T. aestivum*

**Figure 4:**
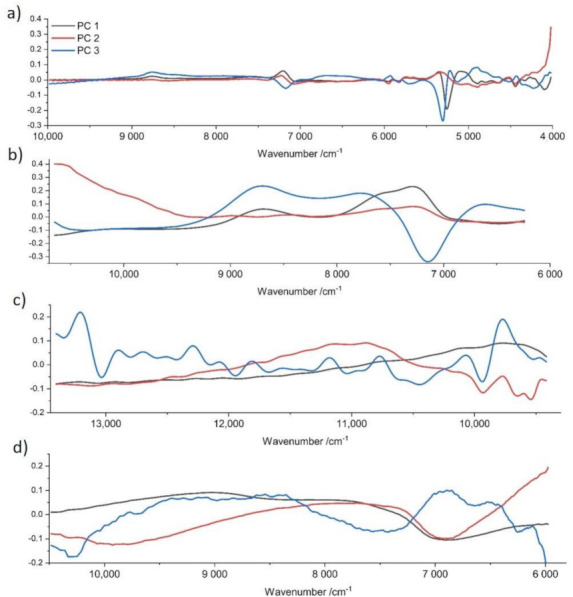
Loading plots of PLS regression models for preprocessed spectra (according to Table 5) including Büchi NIRFlex N-500 (**a**), VIAVI solutions MicroNIR 1700 (**b**), Consumer Physics SCiO (**c**) and Tellspec Enterprise Sensor (**d**).

Eight samples of *T. aestivum* were studied over a time period of 26 days (the first 5 days after harvesting) and spectra were recorded after every three consecutive days. Between measurements, truffles were stored in ideal storage conditions wrapped in separate cellulose wipes in a sealed plastic box. PLS regression models were constructed after spectral pretreatment (Table 5) in order to determine the approximate age of truffles. Corresponding loading plots are represented in Figure 4. Due to the small number of samples, full cross validation was used for model evaluation. To be able to compare the results of the four different NIR devices, the root mean square error of calibration (RMSEC) and validation (RMSEV), and the determination coefficients (R^2^), were calculated and are represented in Table 4.

#### 2.2.5. Feasibility Review—*T. melanosporum* Classification According to the Geographical Origin

All 30 samples of the class *T. melanosporum*, including seven samples from France, six samples from Barcelona (Spain), and 17 samples from Valencia (Spain) were investigated in a separate dataset (pretreatment adopted according to Table 5). Due to the limited number of samples, an attempt was made to create a supervised classification model (LDA) according to the origin of truffle samples.

**Figure 5:**
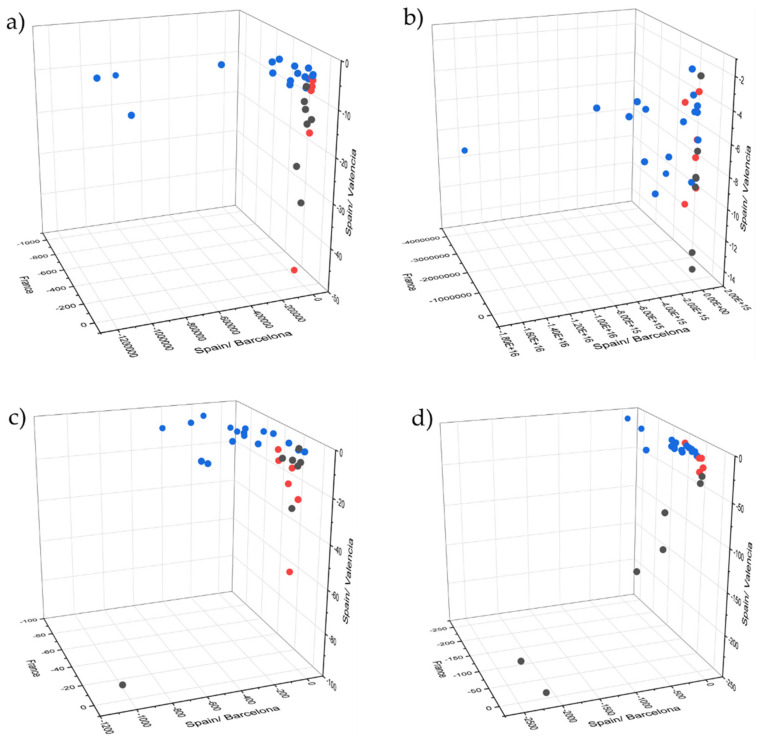
LDA plots of *T. melanosporum* according to the geographical origin (France—black; Spain/Barcelona—red; Valencia—blue) of the samples from France, Barcelona (Spain) and Valencia (Spain) with five applied factors. (**a**) Büchi NIRFlex N-500; (**b**) VIAVI solutions MicroNIR 1700; (**c**) Consumer Physics SCiO; (**d**) Tellspec Enterprise.

Although no clear segregation by geographical origin is displayed in Figure 5, a trend in the sample distribution can be observed for NIRFlex N-500 and Tellspec Enterprise. LDA accuracies of 86.67%, 80.00%, 93.33% and 96.67% were achieved for NIRFlex N-500, MicroNIR 1700, SCiO and Enterprise Sensor, respectively.

## 3. Discussion

In this section, we discuss the results obtained for the classification of fresh truffles, the discrimination between *T. melanosporum* and *T. indicum*, and, furthermore, summarize the output for the quality of truffles.

The results showed that *Tuber aestivum*, *Tuber indicum*, *Tuber melanosporum* and *Tuber uncinatum* can be readily classified, achieving high accuracies. Furthermore, different measurement techniques were applied and validated to identify the optimal compromise between accuracy and applicability for consumers. It was found that stationary measurements of the outer skin achieved better or equivalent results on a rotational platform or a fresh cut. This can be explained by the highly diverse and irregular geometric shapes, which makes it hard to ensure a uniform distance between the sensor and the sample during the spectral acquisition for measurements on the rotational plate. For this reason, results investigating the quality status of truffle were performed using method 1. For stationary measurements, the FT-NIR device NIRFlex N-500 achieved outstanding 100% accuracy for the independent validation set consisting of 42 truffle samples. Slightly worse results were achieved for the handheld NIR-devices, MicroNIR 1700, SCiO and Enterprise Sensor, which correctly predicted 90.5%, 90.5% and 78.6% of samples, respectively.

Particular attention was paid to the discrimination between *Tuber melanosporum* and *Tuber indicum*, which are especially prone to food fraud or adulteration. In this case, diversity in prediction accuracies decreased for benchtop and handheld devices, for which 100% was achieved for NIRFlex N-500, MicroNIR 1700 and SCiO, and 90.5% for the Enterprise Sensor, with regard to measurements of the outer skin. This enables consumers to identify cases of adulteration with high accuracy within seconds using a handheld device that is smaller than a smartphone.

Due to the limited period of time available to store and sell fresh truffles, truffles are frequently frozen and sold as fresh, which can be considered as food adulteration. The loss in quality of frozen truffles has previously been studied and identified via their volatile profile [27]. Discrimination between fresh and frozen truffles using a non-invasive technology, therefore, represents a step forward in the prevention of food fraud.

Near-infrared spectra not only provide the opportunity to discriminate between *Tuber* species, but also to gain information about the quality status of truffles. It was shown that it is possible to a distinguish within each *Tuber* species. Discrimination between fresh and frozen/defrosted truffle samples was undertaken for all handheld devices with sufficient overall accuracies of 85.71% for MicroNIR 1700, 85.71% for SCiO and 88.89% for Tellspec Enterprise sensor. Nevertheless, it was shown that the benchtop FT-NIR device was superior, achieving an overall accuracy of 100%.

In order to make a statement about the degree of freshness and to additionally obtain an overview of the quality of truffles, an attempt was made to predict the approximate date of harvest. Results of the FT-IR device revealed a relatively precise approximation, reaching an accuracy of about 1.5 to 2.5 days. In this case, limitations of the handheld devices were revealed, reaching significantly higher deviations of up to 4.5 days. Loading plots (Figure 4) of the partial least squares regression reveal a strong relation between water bands (10,300, 8333, 6897, 5155 cm^−1^) and the age of the truffle. This fact can be explained by the constant loss of water, despite optimal storage conditions. However, this may also be a reason for the imprecision of the PLS model, as the water content of fresh truffle slightly varies after harvesting due to different environmental conditions at the place of harvest. The region around the O-H deformation and second overtone at about 7142 cm^−1^, which was covered by all devices except SCiO, contributes a significant portion through absorbance by proteins and carbohydrates, although information is superimposed by water. Regions near the visible range (>9000 cm^−1^) contribute less to the regression models, as can be seen especially for SCiO (Figure 3c) whereby the loadings plot show less prominent bands. In this case, principal component three shows rather noisy loadings; hence, fewer components were used for model construction. The spectral region around 4000 cm^−1^ for NIRFlex N-500 (Figure 3a) shows a significant influence, which can be attributed to the increased percentage of lipids caused by the loss of water. Considering the results of the cross validation, an approximate date of harvest can be assumed, although it is impossible to make exact predictions.

*T. melanosporum* achieves varying market prices, depending on the geographical origin of the truffle. Therefore, there is reasonable interest about the place of harvest among consumers. Limited by the low number of samples, a separation according the geographical origin of *T. melanosporum* was provided in this feasibility study. The results presented in full cross validation showed a large range for prediction accuracies depending on the device used, ranging from 86.67% to 96.67%. Although this topic needs to be investigated in more detail, a trend in the right direction was revealed.The results of this study lay the foundation for successful geographical separation of *T. melanosporum* using near-infrared spectroscopy. However, it can be assumed that handheld devices are not suitable for these applications due to a limited wavelength range, limited resolution, and limited sensitivity.

## 4. Materials and Methods

### 4.1. Truffle Samples

Thirty *Tuber melanosporum* samples (Barcelona, Spain; Valencia, Spain; France), 31 *Tuber aestivum* (Bulgaria; Italy) and 30 *Tuber uncinatum* (Italy, France) were freshly obtained by a local truffle trader, guaranteeing species and origin of samples. Thirty-five *Tuber indicum* (China) were bought at a local delicatessen trader. Truffles for classification purpose were directly measured after delivery to ensure the fresh conditions of the samples. After fresh samples were recorded, truffles were frozen at −20 °C for at least 24 h, wrapped in separate cellulose wipes and collected in plastic containers for ideal storage conditions. For discrimination between fresh and frozen/thawed truffle, 15 samples of each species were defrosted to room temperature and measured. Aging studies were representatively performed for *T. aestivum* using a set of eight independent truffles directly measured after delivery (five days after harvesting) and after three consecutive days for a period of 26 days.

### 4.2. Spectroscopic Data Acquisition

**Figure 6:**
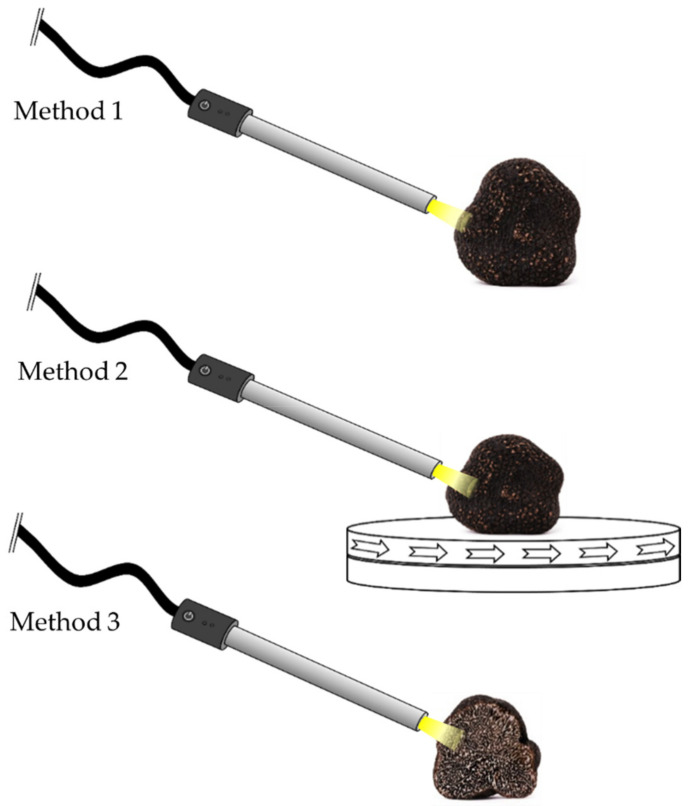
Representative spectral acquisition methods including measurement of the outer skin (method 1), on a rotational device (method 2) and at a fresh cut (method 3).

In order to enable analysis that is applicable for consumers, the performance of three miniaturized near-infrared devices based on dispersive elements acquisition was compared to results obtained by a FT-NIR benchtop spectrometer. Three different measuring techniques were applied for this purpose, according to Figure 6, recording the diffuse reflectance of the outer shell (peridium) (method 1), the outer shell on a rotational device (method 2), and the fruiting body (gleba) at a fresh cut (method 3). For stationary measurements (method 1 and 3), spectra were recorded at three independent and different spots of the sample, providing a representative overview. Method 2 was executed on a platform, rotating with a speed of approximately four rounds per minute. Spectral acquisition time for NIRFlex N-500 (about 35 s) and MicroNIR (about 30 s) was adapted to record multiple rotations. For SCiO and Enterprise Sensor, recording time is limited by the software and therefore not modifiable. For quality monitoring (discrimination fresh/frozen-thawed and aging studies) method 1 was applied.

### 4.3. Near Infrared Spectrometer

For the measurement setup, the samples were placed at a distance of approximately 1 cm from the sensor. Each sample was scanned three times and averaged for each performed methodology. Spectra for MicroNIR1700 and NIRFlex N-500 were obtained after warming up the device, which took approximately 10 and 5 min, respectively. The SCiO and Enterprise Sensor could be readily used.

The FT-NIR benchtop device NIRFlex-500 (Büchi, Flawil, Switzerland) provides illumination using a tungsten halogen lamp and a polarization interferometer with TeO_2_ wedges. A temperature-controlled InGaAs detector operating between 0 and 80 °C provides a spectral range between 4000 and 10,000 cm^−1^ (2500–1000 nm), a resolution of 8 cm^−1^ (interpolated to 4 cm^−1^ by the control software NIR Ware 1.4.3010 (Büchi, Flawil, Switzerland)) and a wavenumber accuracy of ±0.2 cm^−1^. Sixty-four scans were averaged for each measurement. Due to the dimensions of the base unit of 350 × 250 × 450 mm (W × D × H), it is unusable for most applications by consumers; nonetheless, it provides highly accurate reference measurements. The device was equipped with a 1 m fiber optic solid attachment.

The first of the three handheld instruments used was a MicroNIR 1700 (VIAVI, Milpitas, CA, USA). Its weight of only 64 g and dimensions of 45 × 50 mm (W × D) enables on-site investigations in various environments. Control of the instrument is performed using a laptop or tablet, which also serves as source of energy. Spectra were recorded for a 30 ms integration time and 1000 scan count in a spectral range between 6000 and 11,000 cm^−1^ (1667–909 nm) using 125 variables separated by a linear variable filter. Background and reference spectra were acquired after 10 min, under the same conditions. Spectra were automatically transformed into absorbance values by the instrument acquisition software Micro-NIR™ Pro v3.1 (VIAVI solutions, Santa Rosa, CA, USA).

Another recently released handheld instrument, called the Enterprise Sensor, by Tellspec (Tellspec LTD, London, UK), was equally tested in terms of its ability for this application. Two integrated tungsten halogen lamps each having power of 0.7 W provide illumination for scans in diffuse reflectance mode. Measurements were performed using a dispersive linear variable filter (LVF) in combination with an uncooled single element 1 mm InGaAs detector, providing a spectral range from 5900 to 11,100 cm^−1^ (1695–909 nm) by generating 256 datapoints, which results in a spectral resolution of approximately 20 cm^−1^. Reference spectra were recorded after five consecutive measurements using a white reference standard (Spectralon). Spectra are transferred to a smartphone via Bluetooth using the software application DC&M 2 (Tellspec LTD, London, UK).

The performance of a low-cost portable spectrometer SCiO™ (Consumer Physics Inc., Tel-Aviv, Israel) weighing not more than 35 g and having dimensions of 54 × 36.4 × 15.4 mm (W × H × D) was investigated using the SCiO™ Lab online application on a smartphone. Illumination of the sample takes place through an infrared light emitting diode (IRED, i.e., LED) which is split by an optical bandpass filter and detected by a 12-element silicon DAD resolution element. Wavenumbers ranging from 9350 to 13,500 cm^−1^ (1069–741 nm) are detected. The data matrix is uploaded to an online SCiO™ cloud database. To be considered, this device operates in the visible/short-wave NIR in comparison to the previously mentioned devices. Samples were placed about 1 cm from the instrument for spectral acquisition [15,16].

### 4.4. Spectral Processing and Multivariate Data Analysis

#### 4.4.1. Spectral Pretreatments

As a first step, recorded reflectance spectra (Büchi NIRFlex N-500, Consumer Physics SCiO, Tellspec Enterprise Sensor) were transformed from reflectance (R) into absorbance (A) by applying a negative common logarithm (log1/R). Spectra obtained by VIAVI Solutions MicroNIR 1700 were used without conversion as they are presented as absorbance spectra by the control software. Additionally, data obtained by MicroNIR 1700 and Tellspec Enterprise were smoothed prior to any subsequent preprocessing using the Savitzky–Golay (SG) algorithm to reduce high spectral noise. Secondly, different sorts of pretreatment were tested upon the data matrix, including standard normal variate (SNV), detrending, and first- and second-order Savitzky–Golay derivatives with a varying number of smoothing points, in order to reduce additive scattering effects. Wavelength regions were slightly narrowed for all instruments after pretreatment (derivative). Tellspec Enterprise Sensor and VIAVI Solutions MicroNIR 1700 data had to be downsized due to high spectral noise around the boundaries of spectra (Table 5). Three consecutive spectra were reduced as mean spectra in a final step. Pretreatments were carried out using The Unscrambler^®^ X 10.5 (CAMO Software AS., Oslo, Norway) to reduce undesirable scattering effects caused by different textures and geometries of the samples. It was found that each instrument had to be handled differently due to varying regions recorded within the near infrared spectral range. The best working spectral treatments were identified using principal component analysis (PCA) and linear discriminant analysis (LDA) for each individual dataset by applying full cross validation. Table 5 lists the spectral pretreatments applied for the respective datasets of each NIR device.

#### 4.4.2. Multivariate Data Analysis

To be able to identify existing patterns, sample matrices for each instrument were handled separately. Principal component analysis (PCA) was carried out for reduced and pre-processed spectra using Unscrambler^®^ X 10.5 (CAMO Software AS., Oslo, Norway) to obtain information about the latent structure and visualize the data. PCA reduces the dimensionality of the data matrix based on the largest variability, and is considered unsupervised classification [32]. In addition, spectral outliers were identified by applying the algorithm of nonlinear iterative partial least squares (NIPALS).

Linear discriminant analysis (LDA) has been successfully applied to a wide range of problems in food analysis, including adulteration [33]. For this reason, LDA was chosen to discriminate between classes, including species, quality and geographical origin. For this purpose, the algorithm creates linear decision boundaries, which are defined to maximize the ratio of between-class to within-class dispersion. LDA therefore focuses on the dissimilarity between spectra and samples are defined in classes [34].

Kennard–Stone sample selection [35] was applied for datasets using linear discriminant analysis for differentiation between either species or fresh and frozen/thawed samples. Therefore, the sample set was divided into calibration and validation sets by applying a ratio of two-thirds to one-third. For discrimination between the geographical origin of truffles within the species *T. melanosporum*, division into calibration and validation sets was avoided due to the limited sample size. In this case, full cross validation was applied [36].

To determine the approximate time of harvesting, partial least squares regression (PLSR) models were constructed for pretreated spectra (method 1) of *T. aestivum*. PLSR represents a convenient and effective method to relate physicochemical properties, such as the age of truffle, to the absorbance of their corresponding spectra. Preprocessed spectra (according to Table 5) were used to construct the PLSR model including a total number of 10 factors for the NIPALS algorithm in full cross validation.

Spectral outliers were identified investigating the influence plot by considering F-Residuals and Hotelling’s T^2^ with an applied confidence interval of 99%. Due to strong additional scatter effects, spectra vary significantly. Therefore, for measurements of the outer skin, especially for the rotational method (method 2), spectral outliers were identified. A more detailed investigation of outlying spectra was carried out, revealing that samples could nonetheless be classified correctly. Therefore, in order to be widely applicable for the handling of consumers and to produce comparable results for different methods and devices, spectral outliers were not excluded from the dataset.

## 5. Conclusions

This study demonstrated the applicability of near-infrared spectroscopy in combination with chemometric methods to distinguish between the four most frequently consumed edibletruffles. Moreover, a statement about the quality of the truffle can be assumed according to the spectral information. Furthermore, this study identified the advantages and accuracies of three state-of-the-art handheld near-infrared devices that enable consumers to detect food adulteration. Nevertheless, results revealed the superiority of benchtop devices compared to handheld devices, caused by limitations in sensitivity, spectral range and spectral resolution.

Near-infrared spectroscopic measurements do not require special training and are not prone to hazardous chemicals or cost-intensive and elaborate sample preparation. Instead, this approach enables fast, non-invasive and cost-effective quality assurance. Therefore, the results of this study may provide the scientific foundation to expand spectroscopic measurements into the daily life of consumers in order to identify and prevent food adulteration in a broader range of foodstuff.

## Figures and Tables

**Table 1:** Results of Büchi NIRFlex N-500, Consumer Physics SCiO, Tellspec Enterprise Sensor and VIAVI solutions MicroNIR 1700 for classification of fresh truffles, applying all three measurement techniques.

	Büchi NIRFlex N-500		VIAVI Solutions MicroNIR 1700		Consumer Physics SCiO		Tellspec Enterprise	
	LDA ^a^	Pred. ^b^	LDA ^a^	Pred. ^b^	LDA ^a^	Pred. ^b^	LDA ^a^	Pred. ^b^
**Method 1**	100%	42/42	100%	38/42	100%	38/42	96.43%	33/42
**Method 2**	100%	42/42	97.44%	34/42	97.62%	36/42	98.81%	31/42
**Method 3**	96.43%	33/42	98.81%	33/42	100%	38/42	100%	37/42

^a^ Prediction accuracy of LDA for the calibration set (selected by KS sample selection). ^b^ Results for prediction of LDA applied on the validation set of 42 truffles selected by KS sample selection.

**Table 2:** Results of LDA for the classification of *T. indicum* and *T. melanosporum* for all applied devices and methods.

	Büchi NIRFlex N-500			VIAVI Solutions MicroNIR 1700		
	LDA ^a^	T.i. ^b^	T.m. ^c^	LDA ^a^	T.i. ^b^	T.m. ^c^
**Method 1**	100%	13/13	8/8	100%	12/12	9/9
**Method 2**	100%	11/11	10/10	100%	13/13	8/8
**Method 3**	100%	9/9	12/12	100%	12/13	8/8
	**Consumer Physics SCiO**			**Tellspec Enterprise Sensor**		
	**LDA ^a^**	**T.i. ^b^**	**T.m. ^c^**	**LDA ^a^**	**T.i. ^b^**	**T.m. ^c^**
**Method 1**	100%	11/11	10/10	100%	11/11	8/10
**Method 2**	100%	12/12	8/9	100%	9/9	9/12
**Method 3**	100%	13/13	8/8	100%	13/13	8/8

^a^ Prediction accuracy of LDA for the calibration set (selected by KS–sample selection). ^b^ Correctly predicted *T. indicum* (T.i.) samples of the independent validation set. ^c^ Correctly predicted *T. melanosporum* (T.m.) samples of the independent validation set.

**Table 3:** Prediction performance of fresh and frozen/thawed truffle for measurements of the outer shell (method 1).

	Büchi NIRFlex N-500			VIAVI MicroNIR 1700		
Species	LDA. ^a^	Pred. ^b^	Pred. ^c^	LDA. ^a^	Pred. ^b^	Pred. ^c^
***T. aestivum***	96.67%	11/11	4/4	100%	11/11	4/4
***T. indicum***	100%	10/10	5/5	90%	11/11	2/4
***T. melanosporum***	100%	12/12	3/3	100%	12/12	3/3
***T. uncinatum***	93.33%	12/12	3/3	86.67%	7/12	3/3
	**Consumer Physics SCiO**			**Tellspec Enterprise**		
	**LDA. ^a^**	**Pred. ^b^**	**Pred. ^c^**	**LDA. ^a^**	**Pred. ^b^**	**Pred. ^c^**
***T. aestivum***	100%	9/9	6/6	96.67%	9/10	4/5
***T. indicum***	90.00%	9/10	4/5	100%	11/11	3/4
***T. melanosporum***	100%	10/10	5/5	100%	11/11	4/4
***T. uncinatum***	83.33%	8/10	3/5	100%	10/10	4/5

^a^ Prediction accuracy of LDA for calibration set (selected by KS-sample selection). ^b^ Results for predicted fresh truffle. ^c^ Results for predicted frozen/thawed truffle.

**Table 4:** Results for partial least squares regression of all four devices for an independent *T. aestivum* sample set.

Device	RMSE_Cal_ /Days	RMSE_Val_ /Days	R^2^_Cal_	R^2^_Val_	Factor
**Büchi** **NIRFlex N-500**	1.48	2.62	0.9536	0.859	10
**VIAVI solutions** **MicroNIR 1700**	2.67	3.46	0.8486	0.7531	9
**Consumer Physics SCiO**	2.01	3.71	0.8700	0.7165	7
**Tellspec** **Enterprise**	3.91	4.48	0.7212	0.6471	4

**Table 5:** Spectral pretreatments of Büchi NIRFlex N-500, Consumer Physics SCiO, Tellspec Enterprise Sensor and VIAVI solutions MicroNIR 1700.

	Büchi NIRFlex N-500	Consumer Physics SCiO	Tellspec Enterprise Sensor	VIAVI Solutions MicroNIR 1700
**1**	log(1/R)	log(1/R)	log(1/R)	-
**2**	SNV	SNV	SG smoothing Polynomial order: 0 Smoothing points: 7	SG smoothing Polynomial order: 0 Smoothing points: 7
**3**	SG derivative Derivative order: 1 Polynomial order: 2 Smoothing Points: 11	SG derivative Derivative order: 1 Polynomial order: 2 Smoothing points: 11	SNV	SNV
**4**	Reduction	Reduction	SG derivative Derivative order: 1 Polynomial order: 2 Smoothing points: 11	SG derivative Derivative order: 1 Polynomial order: 2 Smoothing points: 11
**5**	Wavenumber region: 9980–4020 cm^−1^	Wavenumber region: 13,422–9390 cm^−1^	Reduction	Reduction
**6**	-	-	Wavenumber region: 10,641–6034 cm^−1^	Wavenumber region: 10,052–6195 cm^−1^

## Data Availability

Not applicable.
